# Towards a biological plausible model of the interaction of long-term memory and working memory

**DOI:** 10.1186/1471-2202-16-S1-P254

**Published:** 2015-12-18

**Authors:** Timo Nachstedt, Florentin Wörgötter, Christian Tetzlaff

**Affiliations:** 1 Third Institute of Physics, Georg-August-Universität, Göttingen, 37077, Germany; 2 Bernstein Center for Computational Neuroscience, Göttingen, 37077, Germany

## 

Learning and memorizing past information are critical features of neural networks. Several models have been proposed to explain the storage and retrieval of different kinds of information [1,2] . These models differ in terms of the types of memories they can store, in terms of the time scales on which they operate, and in terms of the persistence of the stored information. In particular, there are neural models for the long-term memory (LTM) system [2] and other neural models for the working memory (WM) system [1]. While there are some controversies whether LTM and WM are realized by distinct brain regions, there is no doubt about the fact that these two systems are at the same time tightly coupled and interact continuously [3]. However, it is still widely unclear how this interaction is actually realized. Here, we demonstrate a model reproducing and explaining the interaction between LTM and WM such as to exploit the abilities and advantages of the individual components.

WM is most probably realized in the prefrontal cortex (PFC). To capture the diverse dynamics observed in PFC during WM experiments, we choose to model WM by a reservoir computing network [4]. Basically, it consists of a large reservoir of randomly and recurrently connected neurons coupled to the input signals. Due to the huge variety of different signals present in the reservoir, these networks can serve as universal function approximators. However, the influence of past inputs fades away within time scales of seconds to minutes.

The hippocampus, especially its CA3 area, is believed to play a major role in LTM formation on time scales of hours to days. We model its auto-associative capabilities by the well-known idea of cell assemblies [5]. According to this idea, declarative knowledge translates into correlations of neural activities which, in turn, lead to a potentiation of excitatory synapses. This process forms groups of highly interconnected neurons - named cell assemblies. If the synapses in a recurrent network are governed by Hebbian plasticity, cell assemblies emerge in an unsupervised manner based on correlations observed in the network input. Experimental findings indicate that rather abstract or general LTM knowledge does not depend on the hippocampus but is transferred to the medial prefrontal cortex (mPFC). In our model, the representations in the mPFC are the central interface between LTM and WM. It receives signals from both the cell-assembly as well as the reservoir network.

Analyzing the capabilities and characteristics of the proposed system, we train the reservoir to perform certain arithmetic operations within different contexts. During operation, the cell assembly network learns to detect and remember associations between context signals and computed results. This enables the system to remember results of earlier calculations beyond the WM capacity and to reuse them for later tasks. Thus, the system is able to perform computations based on short-term memories of the operands as well as to overcome the limits of the short-term memory by storing computed results into the long-term memory and transferring them back for further usage.
